# *Notes from the Field:* Nationwide Hepatitis E Outbreak Concentrated in Informal Settlements — Namibia, 2017–2020

**DOI:** 10.15585/mmwr.mm6912a6

**Published:** 2020-03-27

**Authors:** Nirma D. Bustamante, Selma Robert Matyenyika, Leigh Ann Miller, Matthew Goers, Puumue Katjiuanjo, Kakehongo Ndiitodino, Emmy-Else Ndevaetela, Undjee Kaura, Kofi Mensah Nyarko, Lilliane Kahuika-Crentsil, Bernard Haufiku, Thomas Handzel, Eyasu H. Teshale, Eric J. Dziuban, Benetus T. Nangombe, Megan G. Hofmeister

**Affiliations:** ^1^Division of Global Health Protection, Center for Global Health, CDC; ^2^Ministry of Health and Social Services, Namibia; ^3^Division of Global HIV & TB, Center for Global Health, CDC; ^4^Division of Viral Hepatitis, National Center for HIV/AIDS, Viral Hepatitis, STD, and TB Prevention, CDC.

In September 2017, Namibia’s Ministry of Health and Social Services (MoHSS) identified an increase in cases of acute jaundice in Khomas region, which includes the capital city of Windhoek. Hepatitis E is a liver disease caused by hepatitis E virus, which is transmitted by the fecal-oral route, causing symptoms consistent with acute jaundice syndrome (*1*). Hepatitis E is rarely fatal; however, the disease can be severe in pregnant women, resulting in fulminant hepatic failure and death (*2*).

On December 14, 2017, MoHSS declared a hepatitis E outbreak in Khomas, which subsequently developed into a nationwide protracted epidemic, centered mainly in informal settlements with poor sanitary conditions. During December 14, 2017–February 2, 2020, a total of 7,247 outbreak-associated hepatitis E cases were reported (Figure). Data on cases were obtained through epidemiologic surveillance, active case finding, and review of health care facility records. Cases were categorized as 1) suspected (acute onset of jaundice or dark urine and pale stools preceded by acute “flu-like” illness and at least one of the following: low grade fever (100.4°F–102.2°F[38°C–39°C]), anorexia, fatigue, nausea or vomiting); 2) epidemiologically linked (suspected case in a person with recent travel to a known outbreak-affected region), or 3) confirmed (acute jaundice with detection of hepatitis E virus immunoglobulin M antibody in serum). Among reported cases, 925 (13%) were suspected, 4,500 (63%) were epidemiologically linked, and 1,822 (24%) were confirmed. All of Namibia’s 14 regions have now detected hepatitis E cases. The majority of cases (59%) occurred in males, and 72% of cases were in persons aged 20–39 years. Sixty-one deaths (0.8%) were reported nationally, including 24 (39%) in pregnant or postpartum women (maternal case fatality rate = 6%). Among all reported cases, 6,068 (84%) were reported from Khomas and Erongo regions, which have large informal settlements. Specifically, 2,677 (37%) cases were reported from three of Namibia’s largest informal settlements, Havana and Goreangab (both in Khomas region), and the Democratic Resettlement Community (in Erongo region). Because of increased rural-urban migration, many low-income workers live in informal settlements with substandard housing and poor sanitation.

**FIGURE:**
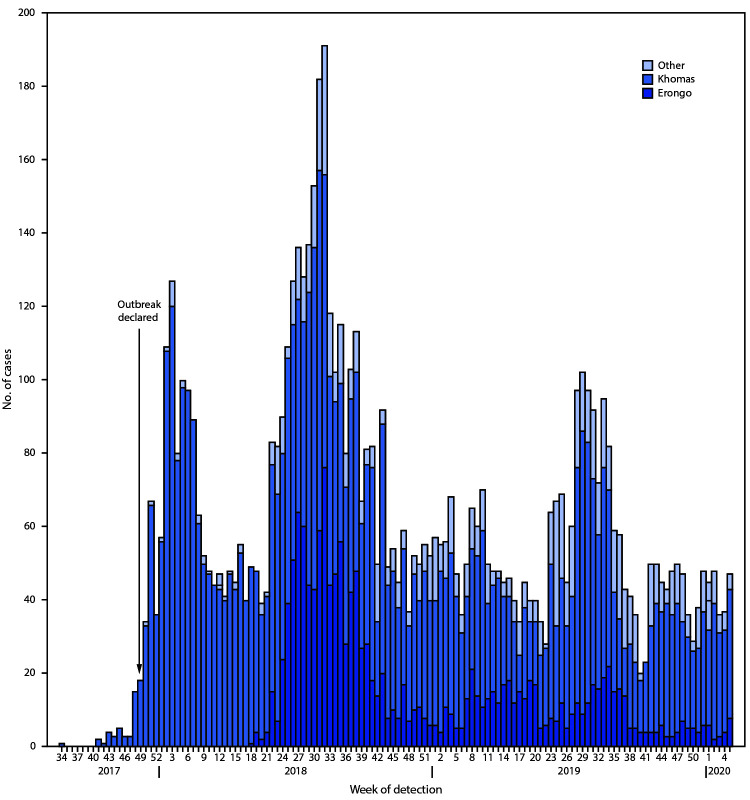
Number of hepatitis E cases (N = 7,247), by week of case detection and region of country* — Namibia, 2017–2020 *Khomas and Erongo regions have large informal settlements and have accounted for 6,068 (84%) of the hepatitis E cases.

CDC has provided technical assistance since the beginning of the outbreak through its Namibia country office. An expanded CDC team was invited to support the national epidemiologic efforts in October 2018. During this response, CDC and international partners helped MoHSS strengthen its surveillance system and data management processes. Activities included standardization of line lists and variables, enhancement of disease-specific surveillance through four national training sessions (totaling 54 staff members), development of effective communication strategies through a reformatted situation report, and supporting implementation of effective outbreak control and prevention measures.

This is the third hepatitis E outbreak described in Namibia since 1983, the largest to date, and the first of nationwide scope (*3*,*4*). The government of Namibia estimates that approximately 40% of households in urban areas are in informal settlements with minimal infrastructure, limited access to latrines and piped water, and poor hygiene (*5*). Although the national population growth rate has been approximately 1.4% per year, in informal settlements the growth rate has been 8%–15% per year (*6*). Improved hand hygiene and sanitation practices and access to safe water are needed to interrupt the transmission of hepatitis E virus in this protracted national outbreak, especially given the high risk of mortality to pregnant women. In Namibia, there are efforts in most affected areas to improve access to and use of latrines through Community-Led Total Sanitation* to address the outbreak.

## Footnotes

* https://www.na.undp.org/content/namibia/en/home/presscenter/articles/2019/launch-of-the-community-led-total-sanitation-campaign-to-fight-h.html.
